# Baseline JAK phosphorylation profile of peripheral blood leukocytes, studied by whole blood phosphospecific flow cytometry, is associated with 1-year treatment response in early rheumatoid arthritis

**DOI:** 10.1186/s13075-017-1278-0

**Published:** 2017-04-11

**Authors:** Krista Kuuliala, Antti Kuuliala, Riitta Koivuniemi, Hannu Kautiainen, Heikki Repo, Marjatta Leirisalo-Repo

**Affiliations:** 1 Bacteriology and Immunology, Helsinki University Hospital and University of Helsinki, Helsinki, Finland; 2 Rheumatology, Helsinki University Hospital and University of Helsinki, Helsinki, Finland; 3 Primary Health Care, Helsinki University Hospital and University of Helsinki, Helsinki, Finland; 4 General Practice, Helsinki University Hospital and University of Helsinki, Helsinki, Finland; 5 Unit of Primary Health Care, Kuopio University Hospital, Kuopio, Finland

**Keywords:** Rheumatoid arthritis, Disease-modifying antirheumatic drug, Janus kinases, Phosphorylation, Blood, Leukocyte, Biomarker

## Abstract

**Background:**

We found recently that baseline signal transducer and activator of transcription 3 phosphorylation in peripheral blood CD4^+^ T cells of patients with early rheumatoid arthritis (RA) is associated with treatment response to synthetic disease-modifying antirheumatic drugs (DMARDs). This prompted us to study the baseline phosphorylation profiles of Janus kinases (JAKs) in blood leukocytes with respect to treatment response in early RA.

**Methods:**

Thirty-five DMARD-naïve patients with early RA provided blood samples for whole blood flow cytometric determination of phosphorylation of JAKs in CD4^+^ and CD8^+^ T cells, CD19^+^ B cells, and CD14^+^ monocytes. Treatment response was determined after 1 year of treatment with synthetic DMARDs, with remission defined as absence of tender and swollen joints and normal erythrocyte sedimentation rate. Exact logistic regression was used to investigate the association of baseline variables with treatment response. Ninety-five percent CIs of means were estimated by bias-corrected bootstrapping.

**Results:**

High JAK3 phosphorylation in CD4^+^ and CD8^+^ T cells, CD19^+^ B cells, and CD14^+^ monocytes and low JAK2 phosphorylation in CD14^+^ monocytes were significantly associated with remission following treatment with synthetic DMARDs.

**Conclusions:**

Baseline JAK phosphorylation profile in peripheral blood leukocytes may provide a means to predict treatment response achieved by synthetic DMARDs among patients with early RA.

## Background

Rheumatoid arthritis (RA) is a chronic autoimmune disease characterized by inflammatory synovitis; articular destruction; extra-articular manifestations; and, when not responding to treatment satisfactorily, progressive disability and increased mortality. The disease is heterogeneous, and its pathogenetic mechanisms are not clear, but they are known to involve both genetic and environmental factors as well as innate and adaptive immunity [1]. Synthetic disease-modifying antirheumatic drugs (sDMARDs) are the first treatment option in untreated RA, used either as monotherapy or in various combinations [2–4]. Recently, highly selective immunological agents have been developed for the treatment of RA, including biological disease-modifying antirheumatic drugs (DMARDs), especially blockers of inflammatory cytokines and immune cell surface receptors [5], and agents inhibiting intracellular signal transduction pathway members. As to the latter, Janus kinases (JAKs) are tempting targets because they are required for classical immune cytokine receptor signal transduction and consequently are involved in the pathophysiology of several inflammatory and immune disorders [6, 7]. Despite the multiple drug options for treatment of RA, the lack of reliable predictive biomarkers poses a challenge to finding the most effective and suitable treatment for each individual rapidly, preferably within 3–4 months from the onset of symptoms [8].

A few years ago, we started to study the phosphorylation of the signal transducer and activator of transcription (STAT) transcription factors as biomarkers of disease activity and treatment response in RA [9]. We found that baseline phosphorylation of STAT3 in whole blood leukocytes is associated with the treatment response achieved by use of sDMARDs [10]. In the present study, we made progress in studying the JAK-STAT pathway and aimed at elucidating if JAK phosphorylation levels are able to distinguish DMARD-naïve patients with RA who will respond well to sDMARDs from those who will require still other treatment options. Using a phosphospecific whole blood flow cytometric method, we measured the baseline phosphorylation levels of all known JAKs (i.e., JAK1, JAK2, JAK3, and tyrosine kinase 2 [Tyk2]) in whole blood T cells, B cells, and monocytes of patients with early untreated RA.

## Methods

### Subjects

The study included 35 patients diagnosed with early RA at the Division of Rheumatology, Helsinki University Central Hospital, Helsinki, Finland, between April 2012 and April 2013. The patients fulfilled the American College of Rheumatology/European League Against Rheumatism 2010 classification criteria [11]. At study entry, the patients had received no DMARDs or oral glucocorticoids. Twenty-one healthy volunteers (62% women; mean age 47 years [SD 13 years]) with no infectious symptoms or history of autoimmune disease and derived from hospital and laboratory staff served as reference subjects. All subjects were white Finnish persons residing in the Helsinki metropolitan area. The study protocol was approved by the ethical review board of the Joint Authority for the Hospital District of Helsinki and Uusimaa, and written informed consent was obtained from each patient.

### Clinical evaluation

A comprehensive clinical and laboratory evaluation was undertaken at entry concomitant to blood sampling. Joints were evaluated for swelling and pain (66 and 68 joints, respectively). Patient global assessment of disease activity was recorded on a 100-mm visual analogue scale. Laboratory measurements, including white blood cell count, erythrocyte sedimentation rate (ESR), and plasma C-reactive protein (CRP) level, were logged. The 28-joint Disease Activity Score (DAS28) was calculated [12]. After a mean 12-month follow-up, the patients were reexamined. Patients with no tender or swollen joints (68 and 66 joints, respectively) and normal ESR were considered to be in remission.

### Blood samples

At study entry, an 8-ml blood sample was taken by venipuncture from the antecubital vein into a Falcon polypropylene tube (BD Biosciences, Bedford, MA, USA) supplemented with 800 μl of pyrogen-free acid citrate dextrose solution A (ACD-A; Baxter Healthcare Ltd, Thetford, UK). Cells were prepared for flow cytometry within 3 h of blood sampling.

### Antibodies

The following antibodies were purchased from BD Biosciences (San Jose, CA, USA): fluorescein isothiocyanate (FITC)-conjugated anti-CD14 (MφP9), anti-CD4 (SK3), and anti-CD19 (SJ25C1); phycoerythrin (PE)-conjugated anti-CD3 (SP34-2); peridinin chlorophyll protein complex (PerCP)-conjugated anti-CD3 (SK7); Alexa Fluor 647-conjugated anti-CD8 (RPA-T8) mouse anti-human monoclonal antibodies; and PE-conjugated anti-rabbit secondary antibody. Anti-phosphorylated (anti-p)-JAK1 (PhosphoDetect Tyr1022/1023) rabbit anti-human antibody was purchased from EMD Millipore (Darmstadt, Germany). The following antibodies were purchased from Santa Cruz Biotechnology (Dallas, TX, USA): anti-pJAK2 (Tyr1007/Tyr1008), anti-pJAK3 (Tyr980), anti-pTyk2 (Tyr1054) rabbit anti-human antibodies, and PerCP-conjugated anti-rabbit secondary antibody. The amounts of the antibodies were optimized, and their compatibility was assured in preliminary examinations.

### Preparing leukocytes for flow cytometry

Blood leukocytes were prepared for flow cytometry using a protocol and buffers by BD Biosciences [13]. Aliquots (50 μl) of blood were deposited into 12 polystyrene tubes (BD Biosciences). Four tubes were supplemented with anti-CD14-FITC, four with anti-CD4-FITC and anti-CD8-Alexa Fluor 647, and four with anti-CD19-FITC for surface marker staining of monocytes, CD4^+^/CD8^+^ T cells, and B cells, respectively. Following a 15-minute incubation at 37 °C, leukocytes were fixed and erythrocytes lysed by adding 1× Lyse/Fix Buffer (BD Biosciences). After being pelleted, leukocytes were permeabilized and washed by incubating them twice in 1× Perm/Wash Buffer (BD Biosciences) at room temperature protected from light for 15 minutes. Cells were pelleted and resuspended in Perm/Wash Buffer. One tube each containing anti-CD14, anti-CD4 and anti-CD8, and anti-CD19 were supplemented with either anti-pJAK1, anti-pJAK2, anti-pJAK3, or anti-pTyk2. The tubes were incubated at room temperature protected from light for 1 h, and then cells were pelleted and resuspended in Perm/Wash Buffer. Tubes containing the anti-pJAK1, anti-pJAK2, or anti-pTyk2 were supplemented with PE-conjugated secondary antibody, and tubes containing anti-pJAK3 were supplemented with PerCP-conjugated secondary antibody. The anti-CD4- and anti-CD8-containing tubes, as well as the anti-CD19-containing tubes, were also supplemented with the T-cell marker antibody anti-CD3-PerCP (for anti-pJAK1, anti-pJAK2, and anti-pTyk2 tubes) or anti-CD3-PE (for anti-pJAK3 tubes). Following incubation at room temperature protected from light for 40 minutes, cells were washed in Perm/Wash Buffer and suspended in Stain Buffer (BD Biosciences). The samples were kept on ice for a maximum of 3 h until flow cytometric acquisition.

### Flow cytometry

Flow cytometric data were acquired using a BD FACSCanto II flow cytometer and analyzed with BD FACSDiva software (BD Biosciences), as described previously [14]. CD3^+^CD4^+^ and CD3^+^CD8^+^ T lymphocytes, CD19^+^ B lymphocytes, and CD14^+^ monocytes were identified, and pJAK1-PE, pJAK2-PE, pJAK3-PerCP, and pTyk2-PE fluorescence intensities of the respective cell populations were determined as relative fluorescence units (RFU).

### Statistical analysis

The data are presented as means with SDs and numbers of patients with percentages, and groups were compared using a permutation test or chi-square test. Exact logistic regression was used to investigate crude and adjusted relationships of the study entry variables to remission at 12 months. The 95% CIs of means were estimated by bias-corrected bootstrapping (5000 replications). pJAK1-PE, pJAK2-PE, pTyk2-PE, and pJAK3-PerCP fluorescence intensities are presented as standardized fluorescence intensities (mean 0, SD 1) of RFU. Correlation coefficients were calculated by the Spearman method using Šidák-adjusted probabilities. The Stata 14.1 statistical software package (StataCorp, College Station, TX, USA) was used for the analyses.

## Results

### Treatment and outcome

The study included 35 DMARD-naïve patients with recent-onset RA (Table 1). After blood sampling, 33 patients started sDMARD therapy (methotrexate, sulfasalazine, hydroxychloroquine) according to the Finnish national care guidelines [4] (Table 1). In addition, 16 patients (46%) started a course of low-dose (≤10 mg/day) oral prednisone. Intra-articular glucocorticoid injections were also applied to treat swollen joints according to the Finnish national guidelines [4].

**Table 1:** Baseline characteristics of the patients categorized according to remission at 12-month follow-up

Variable	All patients (*n* = 35)	Remission at follow-up		*p* Value^a^
		No (*n* = 15)	Yes (*n* = 20)	
Age, years, mean (SD)	55 (14)	57 (15)	53 (14)	0.43
Female sex, *n* (%)	28 (80%)	15 (75%)	13 (87%)	0.39
Rheumatoid factor-positive, *n* (%)	26 (74%)	10 (67%)	16 (80%)	0.37
Anti-citrullinated protein antibody-positive, *n* (%)	27 (77%)	10 (67%)	17 (85%)	0.20
Smokers, *n* (%)	8 (23%)	5 (33%)	3 (15%)	0.20
Duration of symptoms, months, mean (SD)	6 (7)	6 (10)	6 (4)	0.89
Number of tender joints, 0–68, mean (SD)	7 (6)	10 (7)	6 (5)	0.040
Number of swollen joints, 0–66, mean (SD)	6 (5)	6 (4)	7 (6)	0.78
Erythrocyte sedimentation rate, mm/h, mean (SD)	19 (15)	22 (18)	16 (13)	0.32
Plasma C-reactive protein, mg/L, mean (SD)	7 (7)	6 (3)	8 (9)	0.41
White blood cell count, ×10^9^/L, mean (SD)	6.9 (2.4)	7.5 (3.1)	6.5 (1.6)	0.23
Patient global assessment, 0–100-mm VAS, mean (SD)	40 (25)	54 (24)	31 (21)	0.0073
DAS28 score, mean (SD)	3.63 (1.14)	4.08 (1.19)	3.29 (1.01)	0.040
Number of DMARDs started, mean (range)	2.2 (0–3)	2.1 (1–3)	2.3 (0–3)	0.72
Single DMARD, *n*	4	2	2	
Combination of two DMARDs, *n*	13	9	4	
Combination of three DMARDs, *n*	16	4	12	
None, *n*	2	0	2	

*Abbreviations: VAS* Visual analogue scale, *DAS28* 28-Joint Disease Activity Score, *DMARD* Disease-modifying antirheumatic drug

^a^ Significance of difference between No and Yes groups calculated using permutation test or chi-square test

During follow-up (mean duration 12 months, SD 3.6 months), the drug treatment was modified when appropriate, targeting remission. After follow-up, 33 patients (91%) were on sDMARDs, including 3 patients (9%) on oral prednisone. Twenty patients (57%) were in remission (no swollen or tender joints, and normal ESR). No patients were on biological DMARDs. The patients in remission started with a lower DAS28 score, lower number of tender joints, and lower patient global assessment at study entry (Table 1).

### Phosphorylation levels of JAKs

**Fig. 1:**
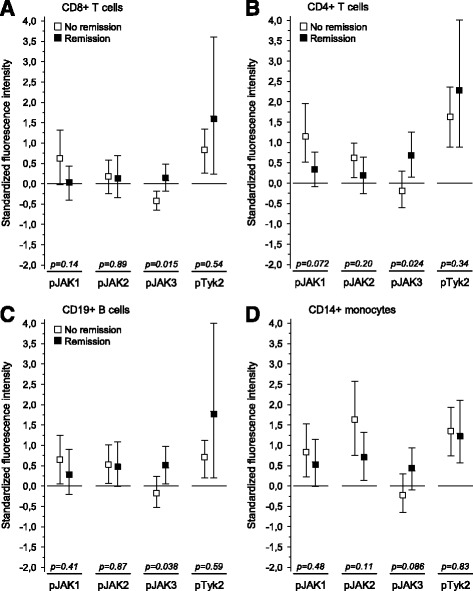
Baseline Janus kinase phosphorylation (pJAK) profiles of peripheral blood **a** CD8+ T cells﻿, **b** CD4+ T cells, **c** CD19+ B cells, and **d** CD14+ monocytes according to remission at 12-month follow-up. *Squares* indicate group means, and *whiskers* indicate group 95% CIs. The fluorescence intensities are standardized to controls (mean 0 and SD 1). *Horizontal lines* show controls’ means. Remission groups are compared using a permutation test

Baseline phosphorylation of levels of JAKs were compared between the patients who subsequently were in remission after 12-month follow-up and the patients who were not (Fig. 1). JAK3 phosphorylation in CD8^+^ and CD4^+^ T cells and CD19^+^ B cells was found to be higher in the patients who achieved remission than in those who did not (*p* values of 0.015, 0.024, and 0.038, respectively). No correlations were found between baseline JAK phosphorylation levels and plasma ESR or plasma CRP levels (data not shown).

In order to further elucidate the predictive value of JAK phosphorylation in each cell population, crude and adjusted exact logistic regression models of the baseline phosphorylation levels of JAK1, JAK2, JAK, and Tyk2 were constructed (Table 2). In the models adjusted for anti-cyclic citrullinated peptide, number of DMARDs started at entry, smoking, and white blood cell count, high phosphorylation level of JAK3 was associated with remission in all leukocyte types studied. In addition, low JAK2 phosphorylation level in CD14^+^ monocytes at baseline emerged to be associated with remission.

**Table 2:** ORs for remission after 1 year of follow-up

Model	Variable	CD8^+^ T cells		CD4^+^ T cells		CD19^+^ B cells		CD14^+^ monocytes	
		OR (95% CI)	*p* Value	OR (95% CI)	*p* Value	OR (95% CI)	*p* Value	OR (95% CI)	*p* Value
Unadjusted	pJAK1	0.57 (0.25–1.18)	0.14	0.47 (0.18–1.03)	0.053	0.75 (0.36–1.49)	0.42	0.78 (0.38–1.56)	0.49
	pJAK2	0.96 (0.48–1.89)	0.90	0.63 (0.30–1.26)	0.20	0.95 (0.48–1.88)	0.88	0.55 (0.25–1.10)	0.092
	pJAK3	3.01 (1.20–9.55)	0.017	2.40 (1.08–6.11)	0.032	2.19 (1.03–5.32)	0.044	1.84 (0.91–4.00)	0.094
	pTyk2	1.34 (0.64–3.24)	0.49	1.23 (0.61–2.84)	0.61	1.46 (0.65–3.78)	0.44	0.92 (0.46–1.82)	0.80
Adjusted^a^	pJAK1	0.42 (0.13–1.14)	0.083	0.46 (0.16–1.09)	0.077	0.68 (0.30–1.49)	0.32	0.84 (0.36–1.94)	0.70
	pJAK2	0.97 (0.46–2.1)	0.94	0.53 (0.25–1.20)	0.14	0.66 (0.29–1.42)	0.29	0.42 (0.16–0.96)	0.034
	pJAK3	3.87 (1.33–16.65)	0.009	2.73 (1.16–7.90)	0.017	2.87 (1.11–8.87)	0.029	2.22 (1.01–5.31)	0.045
	pTyk2	1.40 (0.64–3.25)	0.45	1.32 (0.62–3.05)	0.52	1.47 (0.63–3.47)	0.44	0.92 (0.45–1.93)	0.83

*pJAK* Phosphorylated Janus kinase, *Tyk* Tyrosine kinase

^a^ The models are adjusted for anti-citrullinated protein antibody, number of disease-modifying antirheumatic drugs at entry, smoking, and white blood cell count. ORs are calculated per 1 SD

Tyk2 phosphorylation level was higher in patients than in healthy reference subjects in all leukocyte types studied (Table 3): CD4^+^ T cells (*p* = 0.0014), CD8^+^ T cells (*p* = 0.037), CD19^+^ B cells (*p* = 0.049), and CD14^+^ monocytes (*p* < 0.001). This indicated constitutive activation of Tyk2 in the patients’ circulating leukocytes. Also, JAK2 phosphorylation of CD14^+^ monocytes was higher in patients than in healthy reference subjects (*p* = 0.011) (Table 3).

**Table 3:** Baseline phosphorylatad Janus kinase fluorescence intensities in peripheral blood leukocytes of patients and healthy reference subjects

Fluorescence	Cell type	Patients (*n* = 35)	Healthy control subjects (*n* = 17)	*p* Value
pJAK1-PE	CD4^+^ T	385 (92)	335 (75)	0.054
	CD8^+^ T	387 (89)	365 (79)	0.41
	CD19^+^ B	549 (151)	497 (121)	0.23
	CD14^+^ Mo	863 (240)	737 (193)	0.06
pJAK2-PE	CD4^+^ T	4443 (1446)	3885 (1484)	0.20
	CD8^+^ T	4282 (1792)	4016 (1761)	0.62
	CD19^+^ B	7675 (3026)	6340 (2680)	0.12
	CD14^+^ Mo	6571 (2703)	4727 (1674)	0.011
pJAK3-PerCP	CD4^+^ T	823 (197)	773 (163)	0.37
	CD8^+^ T	1350 (283)	1388 (396)	0.69
	CD19^+^ B	1040 (214)	994 (213)	0.47
	CD14^+^ Mo	2035 (442)	1976 (385)	0.65
pTyk2-PE	CD4^+^ T	380 (144)	297 (41)	0.001
	CD8^+^ T	342 (128)	287 (43)	0.037
	CD19^+^ B	361 (177)	295 (51)	0.049
	CD14^+^ Mo	1118 (342)	827 (228)	<0.001

*Abbreviations: pJAK* Phosphorylated Janus kinase, *Mo* Monocyte, *PE* Phycoerythrin, *PerCP* Peridinin chlorophyll protein complex, *Tyk* Tyrosine kinase

The fluorescence intensities are shown as means (SDs) in relative fluorescence units, with *p* values calculated using a permutation test

## Discussion

The results of the present study show that high baseline JAK3 phosphorylation in all peripheral blood leukocyte subtypes studied (i.e., CD4^+^ and CD8^+^ T cells, B cells, and monocytes) is associated with remission achieved by sDMARDs among patients with early untreated RA. The JAK signaling profile of the patients who turned out as good sDMARD responders also included low JAK2 phosphorylation in CD14^+^ monocytes. Although there is evidence of JAK activation within inflamed joints observed in a few studies using animal models [15] or synovial cells from patients with RA [16–18], there are no prior studies, to the best of our knowledge, on the phosphorylation of JAKs in peripheral blood leukocytes of patients with RA. Consequently, the potential of the JAK phosphorylation profile in leukocytes as a predictor of treatment response in RA has not been studied before.

There are a number of possible explanations for the association we found between baseline JAK3 phosphorylation level and treatment response. First, the sDMARDs used can be effective in downregulating JAK3-involving inflammatory pathways, although the detailed mechanisms remain to be elucidated. In this context, it may be noteworthy that JAK3 cannot be replaced by other JAKs in transducing signals from the receptors of interleukin (IL)-2, IL-4, IL-7, IL-9, IL-15, and IL-21 (γc receptors) [7]. These cytokines are among those whose levels in peripheral blood are affected by DMARDs [19–22], which may represent a disease-modifying mechanism involving JAK3 signaling. Second, the anti-inflammatory or regulatory processes that are also triggered by JAK3 can possibly facilitate the amelioration of RA. It has been observed that JAK3 signaling limits T-helper cell type 17 differentiation in CD4^+^ T cells [23], maintains circulating quiescent T cells [24], and negatively regulates the production of inflammatory cytokines in innate immune cells [25, 26]. In fact, in developing kinase inhibitor therapies for the treatment of RA, the challenge is to halt the pathogenic effects while preserving the homeostatic and advantageous effects of the kinases [27].

The association between low JAK2 phosphorylation in monocytes and good treatment response may reflect the critical roles of JAK2 in innate immune responses. It has been demonstrated in monocytic cells that JAK2 inhibition blunts the production of both early and late inflammatory mediators [28], and that the prolonged effect of interferon-γ on lipopolysaccharide-induced tumor necrosis factor production is apparently mediated by JAK2 [29]. It is noteworthy that the JAK inhibitor tofacitinib has high affinity for JAK3, but it also inhibits JAK1 and, to a lesser extent, JAK2, and also STAT1 and STAT3 [6]. Thus, the efficacy of tofacitinib in treating RA [6, 7, 30] may actually be due to its effects on target molecules other than JAK3. It has also been reported on the basis of in vitro studies on rheumatoid synovial fibroblasts that JAK3-selective inhibition is insufficient to control the proinflammatory cascade, and inhibition of JAK1 and JAK2 may be needed instead [31].

It should be noted that because the patients’ JAK3 phosphorylation levels overlapped with those of healthy reference subjects, they cannot serve as diagnostic markers for RA. The phenomenon in which levels of a marker distinguish patients with RA according to divergent outcomes while not distinguishing patients from healthy control subjects has recently been described for other potential markers, including microRNAs [32]. Hence, JAK3 phosphorylation may serve as a surrogate marker, and the possibility remains that markers that discriminate patients with RA from healthy individuals will be discovered along the same signaling pathway.

We also observed that, in contrast to the other JAKs, Tyk2 phosphorylation in lymphocytes and monocytes of patients with RA was significantly higher than in those of healthy reference subjects. Because this phosphorylation was not associated with measures of disease activity or treatment response, the context of the finding remains elusive. Tyk2, for its part, promotes interferon-γ production [33] and can be activated by IL-13 [34]. Methotrexate treatment (which was an essential sDMARD in our study) reduces the levels of both of these cytokines in patients with RA [19]. So, it is possible that Tyk2 phosphorylation increases along with the inflammatory processes during the development of RA and decreases along with the amelioration achieved by sDMARDs, but its effects are not directly related to clinical manifestations of RA. Interestingly, genetic variants of *Tyk2* have been reported to protect against RA, systemic lupus erythematosus, and possibly other autoimmune diseases, such as inflammatory bowel disease [35]. Accordingly, Tyk2 phosphorylation in patients with early RA (as a consequence of either genetic factors or pathogenetic processes) may be associated with increased risk for development of RA, though not directly correlating to disease activity.

The quite low number of subjects is a limitation of the present study. The strengths of the study include the advantages of the phosphospecific whole blood flow cytometric method we used. Blood samples were easily obtained, and only a small volume of blood (a couple of milliliters) was required. Also, ex vivo phosphorylation due to sample handling was minimized, and the procedure was quick (within hours) and has been evaluated as being applicable for rapid immune status determination before starting immunomodulatory therapy in inflammatory diseases [36]. If our results are confirmed in larger studies, it is possible that JAK3 phosphorylation, either alone or with other markers from, for example, the JAK-STAT pathway, is able to serve as treatment response marker in RA.

## Conclusions

Baseline JAK phosphorylation profile in circulating leukocytes of patients with early untreated RA is associated with treatment response achieved with sDMARDs. High JAK3 phosphorylation level in both CD4^+^ and CD8^+^ T cells, in CD19^+^ B cells, and in CD14^+^ monocytes is a promising predictor of good treatment response to sDMARDs in RA.

## Abbreviations

CRP: C-reactive protein
DAS28: 28-joint Disease Activity Score
DMARD: Disease-modifying antirheumatic drug
ESR: Erythrocyte sedimentation rate
FITC: Fluorescein isothiocyanate
IL: Interleukin
JAK: Janus kinase
Mo: Monocyte
PE: Phycoerythrin
PerCP: Peridinin chlorophyll protein complex
RA: Rheumatoid arthritis
RFU: Relative fluorescence unit
sDMARD: Synthetic disease-modifying antirheumatic drug
STAT: Signal transducer and activator of transcription
Tyk: Tyrosine kinase
VAS: Visual analogue scale
